# Injury and death during the ISIS occupation of Mosul and its liberation: Results from a 40-cluster household survey

**DOI:** 10.1371/journal.pmed.1002567

**Published:** 2018-05-15

**Authors:** Riyadh Lafta, Maha A. Al-Nuaimi, Gilbert Burnham

**Affiliations:** 1 Department of Community Medicine, Al Mustansiriya University, Baghdad, Iraq; 2 National Center for Research and Treatment of Blood Diseases, Baghdad, Iraq; 3 Department of International Health, The Johns Hopkins Bloomberg School of Public Health, Baltimore, Maryland, United States of America; Umeå Centre for Global Health Research, Umeå University, SWEDEN

## Abstract

**Background:**

Measurement of mortality and injury in conflict situations presents many challenges compared with stable situations. However, providing information is important to assess the impact of conflict on populations and to estimate humanitarian needs, both in the immediate and longer term. Mosul, Iraq’s second largest city, was overrun by fighters of the Islamic State of Iraq and Syria (ISIS) on June 4, 2014. In this study, we conducted household surveys to measure reported deaths, injuries, and kidnappings in Mosul, Iraq, both during the occupation of the city by fighters of ISIS and the months of Iraqi military action known as the liberation.

**Methods and findings:**

Mosul was overrun by ISIS forces on June 4, 2014, and was under exclusive ISIS control for 29 months. The military offensive by Iraqi forces, supported by coalition artillery and airstrikes, began on October 17, 2016, in east Mosul and concluded in west Mosul with the defeat of ISIS on June 29, 2017. We conducted a 40-cluster population-based survey as soon as the security forces permitted access for the survey team. The objective of the survey was to measure reported deaths, injuries, and kidnappings in Mosul households during 29 months of ISIS-exclusive control (June 2014–October 2016) and the nine months of Iraqi military action known as the liberation (October 2016–June 2017). In east Mosul, the survey was conducted from March 23 to March 31, 2017, and in west Mosul from July 18 to July 31, 2017. Sampling was based on pre-ISIS population distribution, with revisions made following the extensive destruction in west Mosul. The 1,202 sampled households included 7,559 persons: 4,867 in east Mosul and 2,692 in west Mosul. No households declined to participate. During the time from June 4, 2014, to the time of the survey, there were 628 deaths reported from the sampled households, of which 505 were due to intentional violence, a mortality rate of 2.09 deaths per 1,000 person-months. Over the entire time period, the group with the highest mortality rates from intentional violence was adults aged 20 to 39: 1.69 deaths per 1,000 person-months among women and 3.55 among men. In the 29 months of ISIS-exclusive control, mortality rates among all males were 0.71 reported deaths per 1,000 person-months and for all females were 0.50 deaths per 1,000 person-months. During the nine months of the military liberation, the mortality rates jumped to 13.36 deaths per 1,000 person-months for males and 8.33 for females. The increase was particularly dramatic in west Mosul. The leading cause of reported deaths from intentional violence was airstrikes—accounting for 201 civilian deaths—followed by 172 deaths from explosions. Reported deaths from airstrikes were most common in west Mosul, while reported deaths from explosions were similar on both sides of Mosul. Gunshots accounted for 86 cases, predominantly in west Mosul where ISIS snipers were particularly active. There were 35 persons who were reported to have been kidnapped, almost entirely prior to the military offensive. By the time of the survey, 20 had been released, 8 were dead, and 7 still missing, according to household reports. Almost all of the 223 injuries reported were due to intentional violence. Limitations to population-based surveys include a probable large survivor bias, the reliance on preconflict population distribution figures for sampling, and potential recall bias among respondents.

**Conclusions:**

Death and injuries during the military offensive to liberate Mosul considerably exceeded those during ISIS occupation. Airstrikes were the major reported cause of deaths, with the majority occurring in west Mosul. The extensive use of airstrikes and heavy artillery risks an extensive loss of life in densely populated urban areas. The high probability of survivor bias in this survey suggests that the actual number of injuries, kidnappings, and deaths in the neighborhoods sampled is likely to be higher than we report here.

## Abstract 2

Using household survey data, Gilbert Burnham and colleagues estimate the extent of injury and death during the ISIS occupation of Mosul and its liberation.

## Author summary

**Why was this study done?:**

- The Islamic State of Iraq and Syria (ISIS) controlled Mosul, Iraq’s second largest city, from June 2014 until it was militarily defeated in June 2017.
- No information was available about population events among those living under the control of ISIS or during the military campaign to destroy ISIS.
- This study was undertaken to measure the injuries, deaths, and kidnappings that occurred during the 29 months ISIS controlled Mosul and the impact on households during the nine months of military actions to regain Mosul.

**What did the researchers do and find?:**

- The study sampled 1,202 households in 40 neighborhoods, 25 in east Mosul and 15 in west Mosul, as soon as Iraqi security forces permitted entry of the survey team.
- The mortality rates during the 29 months of exclusive ISIS control were 0.71 and 0.50 reported deaths per 1,000 person-months for males and females, respectively. During the military campaign against ISIS, these mortality rates jumped to 13.36 and 8.33 reported deaths per 1,000 person-months. The increase was particularly dramatic in west Mosul and high among males.
- The leading cause of reported deaths among the 505 who died from intentional violence was airstrikes—accounting for 201 civilian deaths in survey households—followed by 172 deaths from explosions.

**What do these findings mean?:**

- Mortality rates were much higher during the nine months of the military liberation of Mosul than during the 29 months of exclusive ISIS control. The rates for reported deaths were much higher in west Mosul, where house-to-house fighting and aerial and artillery attacks were more intense and population density greater. High mortality rates resulted despite the use of modern precision-targeted ordnance.

## Introduction

Measurement of mortality in conflict situations using epidemiological methods presents many challenges [1]. Numbers of deaths can be counted by observers, as has been done in the Syrian crisis [2]. Such counts, even if meticulously done, risk both substantial undercounting and potential double counting. With extensive displacement, accurate denominators may not be available or based on out-of-date census data, making sampling difficult. Where households are fragmented, key informants may be missing. Even when the household is intact, recall bias may occur. Survival bias, in which households have been destroyed and are not present to report, can cause serious underreporting [3]. Assessing causes of death is also difficult because populations may be unable or unwilling to report causes [4]. Prevailing political opinions may result in findings being maligned or dismissed [5].

Fighters belonging to the Islamic State of Iraq and Syria (ISIS) overran Mosul, Iraq’s second largest city, on June 4, 2014 (Box 1). This caused the flight of Iraqi military forces and perhaps a half-million civilians. Once under ISIS control, the city’s economy rapidly collapsed [6]. Taxes were increased, unemployment rose, services deteriorated, electricity generation stopped completely, and poverty increased substantially. Houses were seized if they belonged to minorities or persons who had fled Mosul. The ability to leave Mosul was severely restricted by ISIS fighters. A rigid set of civil and religious codes were put in place. Dress codes for women became more extreme, requiring even the eyes to be covered with a black gauze. The omnipresent Al Hesba, or morality police, patrolled the city streets, offices, schools, and hospitals, issuing citations to women deemed to be inappropriate in behavior or dress. Health workers were forbidden to speak with persons of the opposite sex, either colleagues or patients. Smoking, picnicking, or watching football videos were punished with fines, flogging, and sometimes death, often meted out with a casual indifference.

**Box 1. Timelines for the ISIS capture and Iraqi recapture of Mosul.**

- December 2013, ISIS and Iraqi government forces clash in Al Anbar province.
- June 4–10, 2014, ISIS forces seize Mosul.
- October 17, 2016, Iraqi military forces attack ISIS-controlled villages east of Mosul.
- November 1, 2016, military forces attack ISIS positions in east Mosul.
- January 24, 2017, east Mosul declared liberated.
- February 19, 2017, military forces attack ISIS positions in west Mosul.
- June 29, 2017, west Mosul declared liberated.

Military preparation to retake Mosul started in 2016. A force was assembled of regular Iraqi military units (Iraqi security forces [ISF]), Popular Militia Units (PMUs), and Kurdish Peshmerga troops. At the start of the military campaign, there were already an estimated 1.2 million displaced persons in the Nineveh governorate, constituting 37.5% of Iraq’s internally displaced persons (IDPs) [7]. An estimated 1 million persons fled Mosul during the liberation campaign [8]. About 1.5 million persons were thought to be still living in Mosul at this time, some having entered Mosul with ISIS.

Military action against ISIS began on October 17, 2016, with attacks in east Mosul (Box 1). Mosul was declared liberated when west Mosul was secured at the end of June 2017. Although the Iraqi government had initially asked residents not to leave the city during the military campaign, some 161,718 persons fled east Mosul and many more from west Mosul [9]. During fighting, residents fleeing would move toward the frontline at great risk, hopefully to be gathered by Iraqi forces for transfer out of Mosul. During the slow military advance into west Mosul, widescale destruction occurred, which obliterated whole neighborhoods. ISIS fighters, retreating from east Mosul, had fortified the area with bombs and booby traps. Civilian populations were used as human shields by ISIS fighters, and those trying to flee were often picked off by snipers [10]. Many who sought safety in the basements of houses were systematically killed. At the same time, there was great concern about the destruction caused by airstrikes on west Mosul [11].

With measurement limitations in mind, we set out to conduct a household survey in Mosul, attempting to improve accuracy and address difficulties previously encountered. The survey objectives were to measure deaths, injuries, and kidnappings during the 29 months of exclusive control by ISIS and during the nine months of Iraqi military action known as the liberation.

## Methods

This population survey was conducted as soon as ISF allowed the survey team entry into Mosul and before large-scale population return began, even though the security situation was still unsettled. Forty neighborhoods or administrative units were randomly selected from Mosul’s established residential administrative units. A typical neighborhood would have contained 200 to 400 dwellings before Mosul’s seizure by ISIS. There were 25 neighborhoods selected on the east side of the Tigris River and 15 selected on the west side, representative of the population distribution of Mosul before seizure by ISIS. There were no data on population shifts or remaining population during ISIS occupation and the subsequent liberation. In each neighborhood, a “start house” was identified by a random method that used a 10-m grid overlay of the selected neighborhood on an aerial map of Mosul. From the start house, 30 adjacent houses were visited, constituting a cluster. Reserve neighborhoods with start houses were identified should the selected neighborhood be inaccessible. The 4 interviewers were physicians with a doctoral degree in community medicine. Three were female, and all were natives of Mosul but had left as ISIS seized Mosul. Training was provided for this specific survey.

The questionnaire was based on those used in several previous Iraqi household surveys. It was adapted for the objectives of this study by the Mosul health workers and Al Mustansiriya University faculty. Pilot testing was carried out in a safer area of Mosul separate from any selected neighborhood and required changes made to the forms and procedures before beginning the survey. The deaths, injuries, and kidnappings sections of the household questionnaire are found in S1 Text. The sample size of 1,200 households was deemed adequate to measure household characteristics of interest in a conflict-affected urban environment and still provide capacity for comparisons, based on several previous surveys in conflict-affected parts of Iraq.

For this survey, a household was defined as a group of people living together, eating from a common kitchen, and living in a structure with a separate entrance from the street. In the case of multihousehold dwellings, a household was considered separate if it had a separate kitchen.

An inclusion requirement was that a household had been present in Mosul during the entire period from June 2014 until the time of the survey. At each house, permission was secured from the head of household. The head of household or the senior female was interviewed. Demographic and household details since June 2014 were recorded. No attempt was made to identify former ISIS supporters or former employees of ISIS. An interview required approximately 1 hour and was conducted in privacy.

After interviewing the start house, interviewers moved to the next dwelling to the right. At intersections, they turned to the right, continuing to the next dwellings. All dwellings were visited consecutively until 30 had been interviewed. Where a dwelling had been destroyed or was unoccupied, the interviewers noted its presence and continued in the same direction to the next occupied dwelling. Interviewer safety was a major concern. The interview teams used caution moving between houses and conducted interviews from midmorning to midafternoon to avoid attention. Sometimes the next housing compound could be entered through a door in a compound wall, thus allowing interviewers to avoid being visible on the street to security forces, although security permissions had been obtained before entering an area. Constant cell phone connections were maintained with other interviewers and with the supervisors. Detailed emergency plans were made for the survey teams about what actions were to be taken if mortar attacks occurred or other physical danger developed. A series of protected locations were identified for emergency shelter. During a mortar attack that did occur, the survey team joined the household they were interviewing in their household shelter.

**Fig 1:**
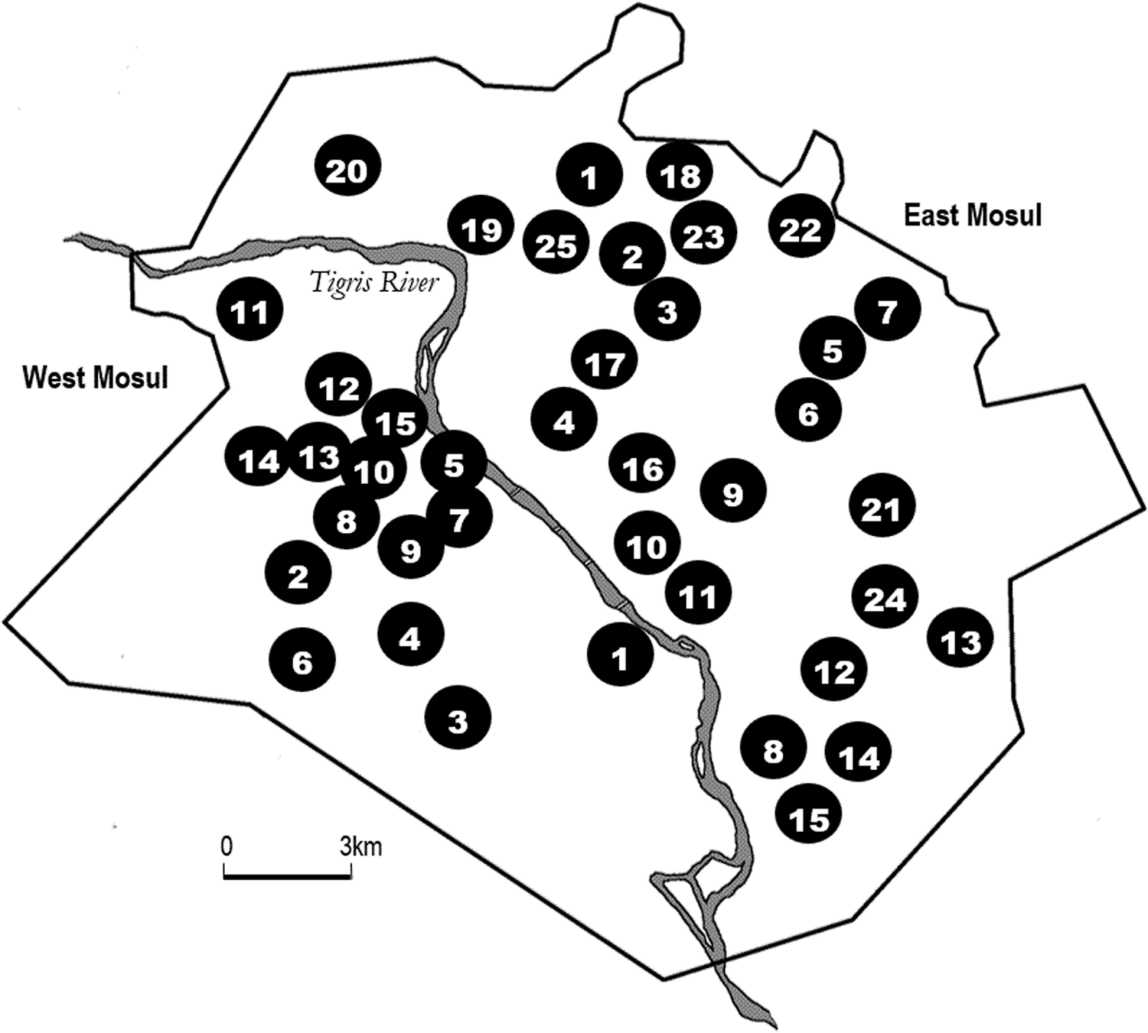
Location of survey clusters in residential areas of east and west Mosul.

The survey of east Mosul was conducted from March 23 to 31, 2017. With the slow military progress, the survey was conducted in west Mosul from July 18 to July 31, 2017. All selected neighborhoods in east Mosul were surveyed as planned. However, the extensive destruction and population shifts in parts of west Mosul required a change in the sampling frame (Fig 1). For this, the team determined which neighborhoods were still inhabited, from which 15 were randomly selected. Of the 5 reserve neighborhoods that were set aside for west Mosul, 2 were eventually used, one because of insecurity and a second to replace a selected neighborhood with few households remaining. The location of the clusters surveyed are listed in Table 1, along with the proportion of houses that were found empty or destroyed in that cluster.

**Table 1:** List of cluster locations with number of empty or destroyed houses in each cluster in brackets.

East Mosul			West Mosul		
1	Al Kindi	[2]	1	Al Jusak	[3]
2	Al Hadbaa	[1]	2	Al Yarmook	[7]
3	Al Baladiyat	[0]	3	Al Mansor	[4]
4	Al Muhandisin	[1]	4	Mosul Al Jadida	[6]
5	Al Kadisiya	[2]	5	Hay Al Shifaa	[25]
6	Al Bakr	[0]	6	Hay Al Risala	[8]
7	Al Zahraa (Saddam)	[2]	7	Al Zanjili	[30]
8	Al Qahira	[3]	8	Al-Warshan	[9]
9	Al Jazaier	[1]	9	Hay Al-Thawraa	[11]
10	Al Dhubbat	[3]	10	Al Rifaaii	[30]
11	Al Baath (Alfuqan)	[1]	11	Msherfaa	[7]
12	Al Wahda	[4]	12	Hawaii-Alkaneesa	[8]
13	Al Entisar	[1]	13	17 Tammus	[10]
14	Dumeez	[1]	14	Ektisadyen	[6]
15	Sumer	[0]	15	Hay Al-Najar	[23]
16	Al Zeraie	[2]			
17	Al Shurta	[5]			
18	Baawiza	[2]			
19	Al Arabi	[1]			
20	Al Rashidiya	[7]			
21	Adan	[8]			
22	Palestine	[1]			
23	Al Siddeck	[1]			
24	Al Methaq	[0]			
25	Al Kafaat (Qairawan)	[1]			

### Analysis

Prior to the data collection, a data analysis plan that included data entry in Baghdad was formulated, with initial frequencies and tabulation to be carried out there. Further analysis would be carried out in Baltimore depending on the results of the initial analysis and discussion. In Baltimore, statistical analysis used Stata version 15 (College Station, TX). The individual exposure times for respondents were calculated beginning from the seizure of control of Mosul by ISIS in June 2014 until the month of interview (March 2017 in east Mosul and July 2017 in west Mosul). For respondents, exposure times reflect the month of entry or exit from the household, notably in the case of death, kidnapping, or birth. Incidence rates for death and injury were calculated from the total number of deaths and/or injuries divided by the total number of person-months contributed using the “stptime” command in Stata, which allowed for variable follow-up time for each individual by dividing the number of failures (i.e., the number of deaths or injuries) by total person-time contributed. Stratified incidence rates were calculated in the same manner individually by age group, geographic area (west/east Mosul), sex, and time period (ISIS occupation versus liberation). For each of these analyses, the number of deaths or injuries in each respective age, sex, area group, or time period was divided by the total number of person-time contributed, then multiplied to estimate rates per 1,000 person-months. To calculate incidence rate ratios (IRRs) comparing death rates between geographic areas (in west versus east Mosul) within sex and age groups, a Poisson model was fit, allowing person-specific time contributed (in person-months). Bootstrapping was used to account for clustering that occurred at the sampling level in calculating CIs [12]. Similar comparison of injury rates was not performed given the limited number and distribution of injuries. Descriptive statistics (numbers and proportions) were also calculated for causes of death and injury, as well as physical location of injuries. Minimal underlying data for this manuscript are deposited publicly in the Humanitarian Data Exchange and can be accessed at https://data.humdata.org/dataset/injury-and-death-during-the-isis-occupation-of-mosul-and-its-liberation.

### Ethical approval

The study received ethical approval from the scientific and technical committee of Al Mustansiriya University, Baghdad. The analysis of deidentified data was exempted by the Institutional Review Board at Johns Hopkins Bloomberg School of Public Health as “not human subjects” research. This study is reported as per the Strengthening the Reporting of Observational Studies in Epidemiology (STROBE) guidelines. The STROBE checklist is found in S1 STROBE Checklist.

## Results

### Household structure

**Fig 2:**
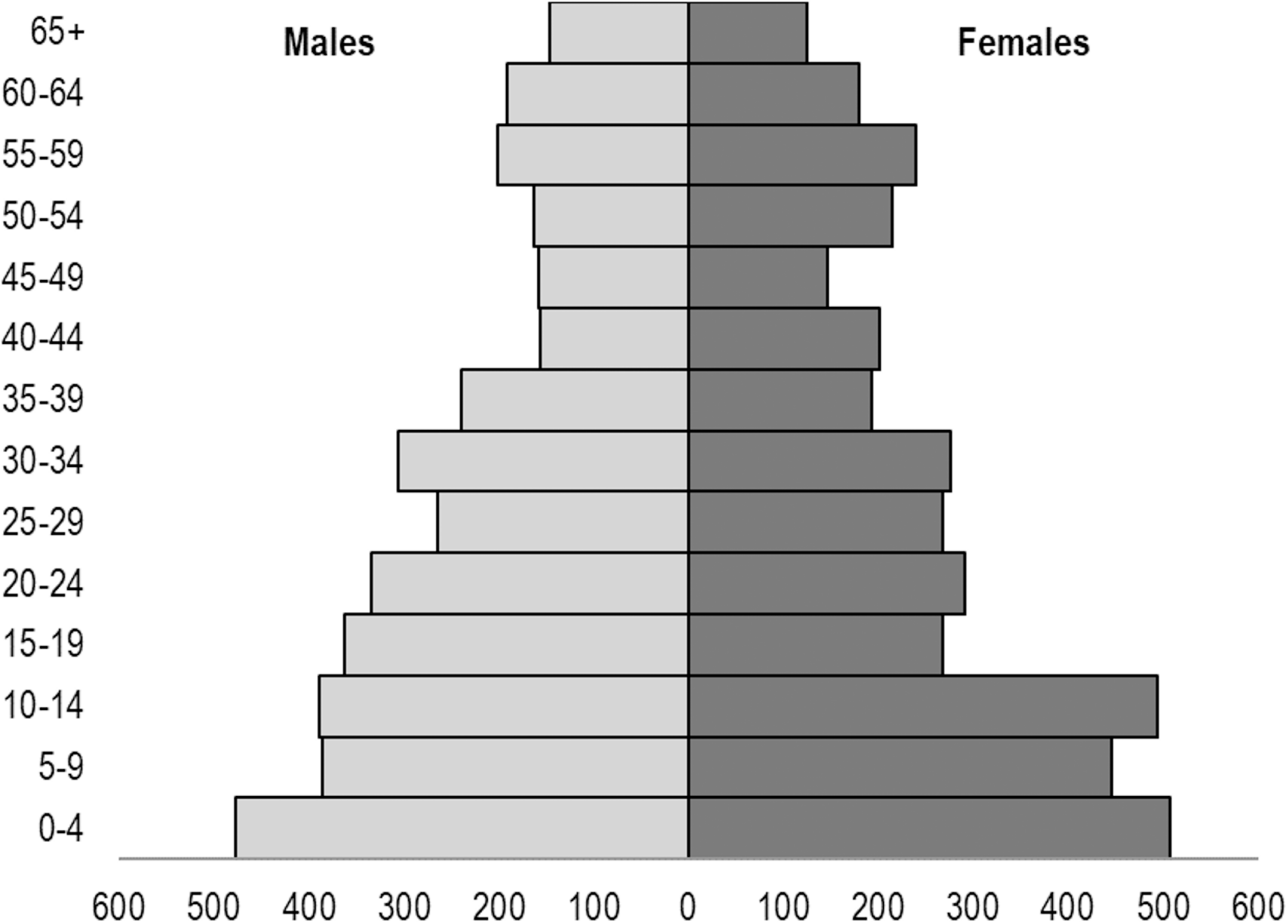
Population pyramid of Mosul study households at the time of survey.

The survey team visited a total of 1,202 Mosul households: 751 in east Mosul and 451 in west Mosul. These 1,202 households contained a total of 7,559 persons (Table 2), with an average household size of 6.5 persons in east Mosul and 6.0 in west Mosul. The age and sex distribution of the sample is shown Fig 2. Almost all dwellings were single-household dwellings. A few structures had several households, usually one of which had moved into a single-family dwelling after their own dwelling had been destroyed. All households approached had been in Mosul during the entire ISIS period; none refused to participate.

**Table 2:** Demographic characteristics of surveyed households.

	East Mosul		West Mosul		All Mosul	
	*N*	(%)	*N*	(%)	*N*	(%)
Young children (0–4 years)	632	(12.99%)	353	(13.11%)	985	(13.03%)
Older children (5–14 years)	1,071	(22.01%)	579	(21.51%)	1,650	(21.83%)
Male adults (15–49 years)	1,172	(24.08%)	650	(24.15%)	1,822	(24.10%)
Female adults (15–49 years)	1,126	(23.14%)	517	(19.21%)	1,643	(21.74%)
Older adults (50+ years)	866	(17.79%)	593	(22.03%)	1,459	(19.30%)
Total	4,867		2,692		7,559	

### Deaths

**Fig 3:**
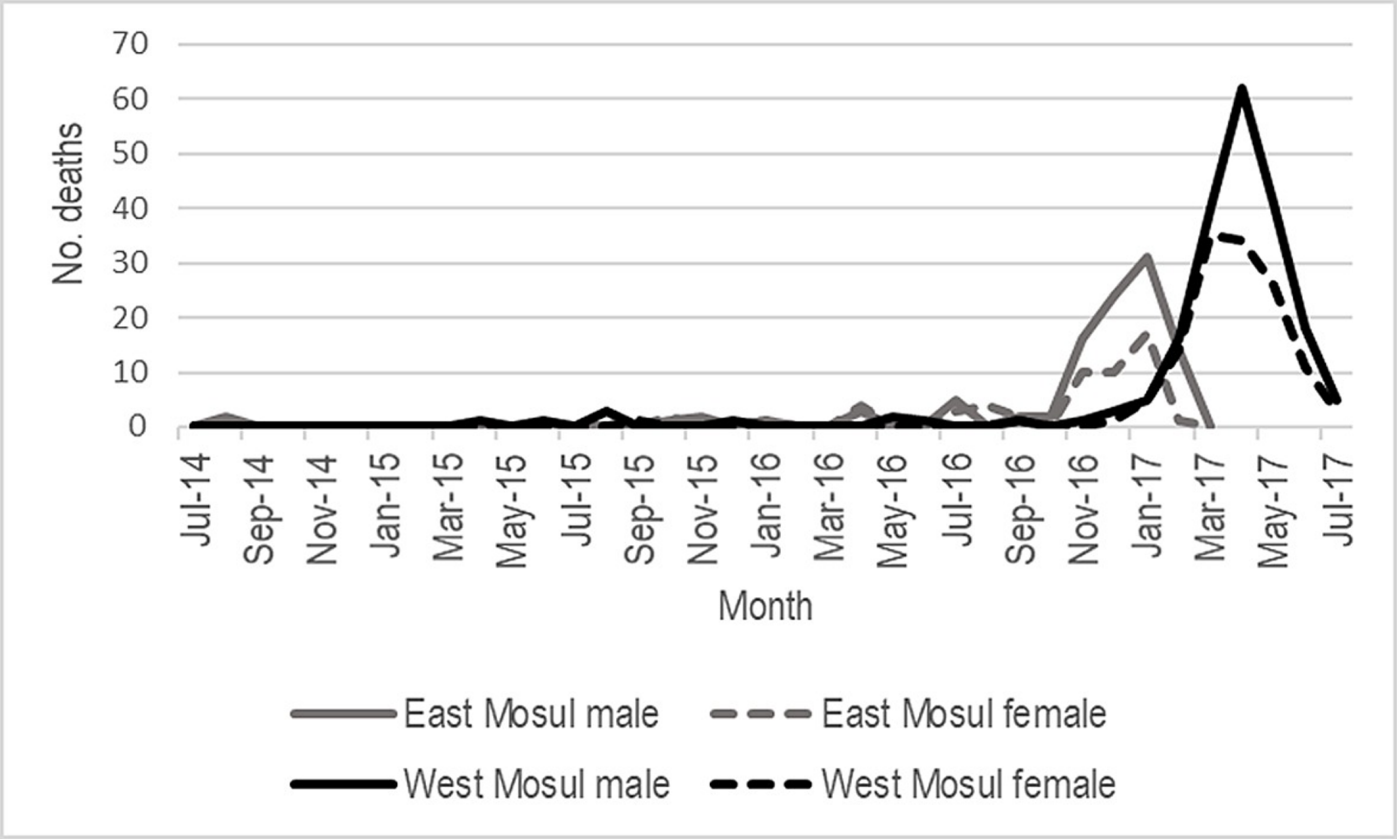
Deaths due to intentional violence by month for east and west Mosul.

**Fig 4:**
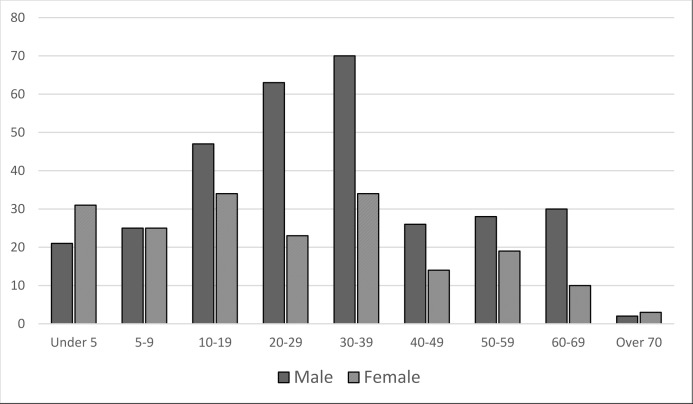
Number of intentional-violence deaths by sex and age over the 36 months.

During the ISIS occupation and the liberation period, there was a total of 241,093 person-months (20,091 person-years) of exposure by the surveyed households in Mosul (Table 3). The full data are shown in person-years in S1 Table. This allows comparison with other events reported in person-years. Exposure in east Mosul was 150,888 person-months (12,574 person-years) and 90,205 person-months (7,517 person-years) in west Mosul. During this time, 628 deaths occurred among survey households in Mosul for an overall rate of 2.61 deaths per 1,000 person-months. Of these 628 deaths, 505 were due to intentional violence, a mortality rate of 2.09 deaths per 1,000 person-months. In east Mosul survey households, the intentional-violence mortality rates for persons under 60 years old ranged between 0.65 for those aged 40 to 59 years to a high of 1.44 for those aged 20 to 39 years. In west Mosul survey households, intentional-violence rates ranged from 2.66 among those 5 to 19 years of age to a high of 4.88 for those aged 20 to 39 years. When comparing the intentional-violence mortality rates between sexes for Mosul survey households as a whole (Table 3), the mortality rates among females was the lowest at 1.23 per 1,000 person-months among those aged 40 to 59 years and highest at 2.37 for children under 5 years. Conversely, among males, intentional-violence mortality rates were lowest among children under 5 years at 1.71 and highest among those in the 20- to 39-year-old age group at 3.55. Deaths occurred principally during liberation (Fig 3). The bimodal pattern in Fig 3 corresponds to the movement of conflict from east to west Mosul. During this time, mortality rates were similar between males and females and between east and west Mosul, although the overall number of deaths was consistently higher among males, especially in the 20- to 39-year-old age group (Fig 4).

**Table 3:** Death and injury rates for west and east Mosul with IRRs for 241,093 person-months of exposure.*

**Deaths**		** **	** **	** **	** **	** **	** **	** **	** **	** **	** **	** **	** **	** **	** **	** **	** **	** **	** **	** **
**All causes**		** **	** **	** **	** **	** **	** **	** **	** **	** **	** **	** **	** **	** **	** **	** **	** **	** **	** **	** **
** **	**West Mosul**						**East Mosul**						**Overall**						**IRR (West/East)**	
	**Males**			**Females**			**Males**			**Females**			**Males**			**Females**			**Males**	**Females**
**Age**	**Person-months**	**Deaths**	**Rate**	**Person-months**	**Deaths**	**Rate**	**Person-months**	**Deaths**	**Rate**	**Person-months**	**Deaths**	**Rate**	**Person-months**	**Deaths**	**Rate**	**Person-months**	**Deaths**	**Rate**	**IRR**	**IRR**
<5	3,979	15	3.77	4,281	25	5.84	8,278	9	1.09	8,809	9	1.02	12,257	24	1.96	13,090	34	2.60	3.47	5.72
5–19	12,859	40	3.11	14,942	38	2.54	24,743	33	1.33	22,943	27	1.18	37,602	73	1.94	37,885	65	1.72	2.33	2.16
20–39	14,404	92	6.39	11,022	37	3.36	23,059	48	2.08	22,708	26	1.14	37,463	140	3.74	33,730	63	1.87	3.07	2.93
40–59	8,772	42	4.79	11,054	30	2.71	13,591	21	1.55	15,676	7	0.45	22,363	63	2.82	26,730	37	1.38	3.10	6.08
60+	4,686	54	11.52	4,206	25	5.94	5,714	28	4.90	5,367	22	4.10	10,400	82	7.88	9,573	47	4.91	2.35	1.45
***Total***	44,700	243	5.44	45,505	155	3.41	75,385	139	1.84	75,503	91	1.21	120,085	382	3.18	121,008	246	2.03	2.95	2.83
**Intentional violence**				** **	** **	** **	** **	** **	** **	** **	** **	** **	** **	** **	** **	** **	** **	** **	** **	** **
** **	**West Mosul**						**East Mosul**						**Overall**						**IRR (West/East)**	
	**Males**			**Females**			**Males**			**Females**			**Males**			**Females**			**Males**	**Females**
**Age**	**Person-months**	**Deaths**	**Rate**	**Person-months**	**Deaths**	**Rate**	**Person-months**	**Deaths**	**Rate**	**Person-months**	**Deaths**	**Rate**	**Person-months**	**Deaths**	**Rate**	**Person-months**	**Deaths**	**Rate**	**IRR**	**IRR**
<5	3,979	15	3.77	4,281	24	5.61	8,278	6	0.72	8,809	7	0.79	12,257	21	1.71	13,090	31	2.37	5.20	7.05
5–19	12,859	40	3.11	14,942	34	2.28	24,743	32	1.29	22,943	25	1.09	37,602	72	1.91	37,885	59	1.56	2.41	2.09
20–39	14,404	90	6.25	11,022	34	3.08	23,059	43	1.86	22,708	23	1.01	37,463	133	3.55	33,730	57	1.69	3.35	3.05
40–59	8,772	40	4.56	11,054	28	2.53	13,591	14	1.03	15,676	5	0.32	22,363	54	2.41	26,730	33	1.23	4.43	7.94
60+	4,686	21	4.48	4,206	12	2.85	5,714.0002	11	1.93	5,367.0002	1	0.19	10,400	32	3.08	9,573	13	1.36	2.33	15.31
***Total***	44,700	206	4.61	45,505	132	2.90	75,385	106	1.41	75,503	61	0.81	120,085	312	2.60	121,008	193	1.59	3.28	3.59
**Nonintentional violence**				** **	** **	** **	** **	** **	** **	** **	** **	** **	** **	** **	** **	** **	** **	** **	** **	** **
** **	**West Mosul**						**East Mosul**						**Overall**						**IRR (West/East)**	
	**Males**			**Females**			**Males**			**Females**			**Males**			**Females**			**Males**	**Females**
**Age**	**Person-months**	**Deaths**	**Rate**	**Person-months**	**Deaths**	**Rate**	**Person-months**	**Deaths**	**Rate**	**Person-months**	**Deaths**	**Rate**	**Person-months**	**Deaths**	**Rate**	**Person-months**	**Deaths**	**Rate**	**IRR**	**IRR**
< 5	3,979	0	0	4,281	1	0.23	8,278	3	0.36	8,809	2	0.23	12,257	3	0.24	13,090	3	0.23	0.00	1.03
5–19	12,859	0	0	14,942	4	0.27	24,743	1	0.04	22,943	2	0.09	37,602	1	0.03	37,885	6	0.16	0.00	3.07
20–39	14,404	2	0.14	11,022	3	0.27	23,059	5	0.22	22,708	3	0.13	37,463	7	0.19	33,730	6	0.18	0.64	2.06
40–59	8,772	2	0.23	11,054	2	0.18	13,591	7	0.52	15,676	2	0.13	22,363	9	0.40	26,730	4	0.15	0.44	1.42
60+	4,686	33	7.04	4,206	13	3.09	5,714.0002	17	2.98	5,367.0002	21	3.91	10,400	50	4.81	9,573	34	3.55	2.37	0.79
***Total***	44,700	37	0.83	45,505	23	0.51	75,385	33	0.44	75,503	30	0.40	120,085	70	0.58	121,008	53	0.44	1.89	1.27
**Injuries**		** **	** **	** **	** **	** **	** **	** **	** **	** **	** **	** **	** **	** **	** **	** **	** **	** **	** **	** **
**All causes**		** **	** **	** **	** **	** **	** **	** **	** **	** **	** **	** **	** **	** **	** **	** **	** **	** **	** **	** **
	**West Mosul**						**East Mosul**						**Overall**						**IRR (West/East)**	
	**Males**			**Females**			**Males**			**Females**			**Males**			**Females**			**Males**	**Females**
**Age**	**Person-months**	**Injuries**	**Rate**	**Person-months**	**Injuries**	**Rate**	**Person-months**	**Injuries**	**Rate**	**Person-months**	**Injuries**	**Rate**	**Person-months**	**Injuries**	**Rate**	**Person-months**	**Injuries**	**Rate**	**IRR**	**IRR**
< 5	3,979	2	0.50	4,281	6	1.40	8,278	0	0	8,809	5	0.57	12,257	2	0.16	13,090	11	0.84	—	2.47
5–19	12,859	31	2.41	14,942	23	1.54	24,743	16	0.65	22,943	18	0.78	37,602	47	1.25	37,885	41	1.08	3.73	1.96
20–39	14,404	27	1.87	11,022	18	1.63	23,059	18	0.78	22,708	6	0.26	37,463	45	1.20	33,730	24	0.71	2.4	6.18
40–59	8,772	13	1.48	11,054	18	1.63	13,591	9	0.66	15,676	1	0.06	22,363	22	0.98	26,730	19	0.71	2.24	25.53
60+	4,686	4	0.85	4,206	4	0.95	5,714.0002	1	0.18	5,367.0002	3	0.56	10,400	5	0.48	9,573	7	0.73	4.88	1.7
***Total***	44,700	77	1.72	45,505	69	1.52	75,385	44	0.58	75,503	33	0.44	120,085	121	1.01	121,008	102	0.84	2.95	3.47

*This table is shown with person-years in S1 Table.

Abbreviation: IRR, incidence rate ratio.

The crude IRR of deaths due to intentional violence in survey households comparing west to east Mosul shows a substantially higher death rate in west Mosul measured in person-months (Table 3). The death rate in west Mosul overall was 3.39 (CI 2.21–5.19) times higher than in east Mosul; 3.28 (CI 2.20–4.88) times higher among males; and among females, 3.59 (CI 1.90–6.78) times higher. Differences in intentional-violence death rates between east and west Mosul households were statistically significant for both males and females (*P* < 0.001). When analyzed by age group, the IRR comparing deaths due to intentional violence in west to east Mosul among females was lowest in the 5- to 19-year-old age group (IRR = 2.09) and highest among those over 60 years (IRR = 15.31). Among males, the highest IRR observed was also among those aged 40 to 59 years (IRR = 4.43) and lowest among those over 60 years old (IRR = 2.33).

### Causes of death

**Fig 5:**
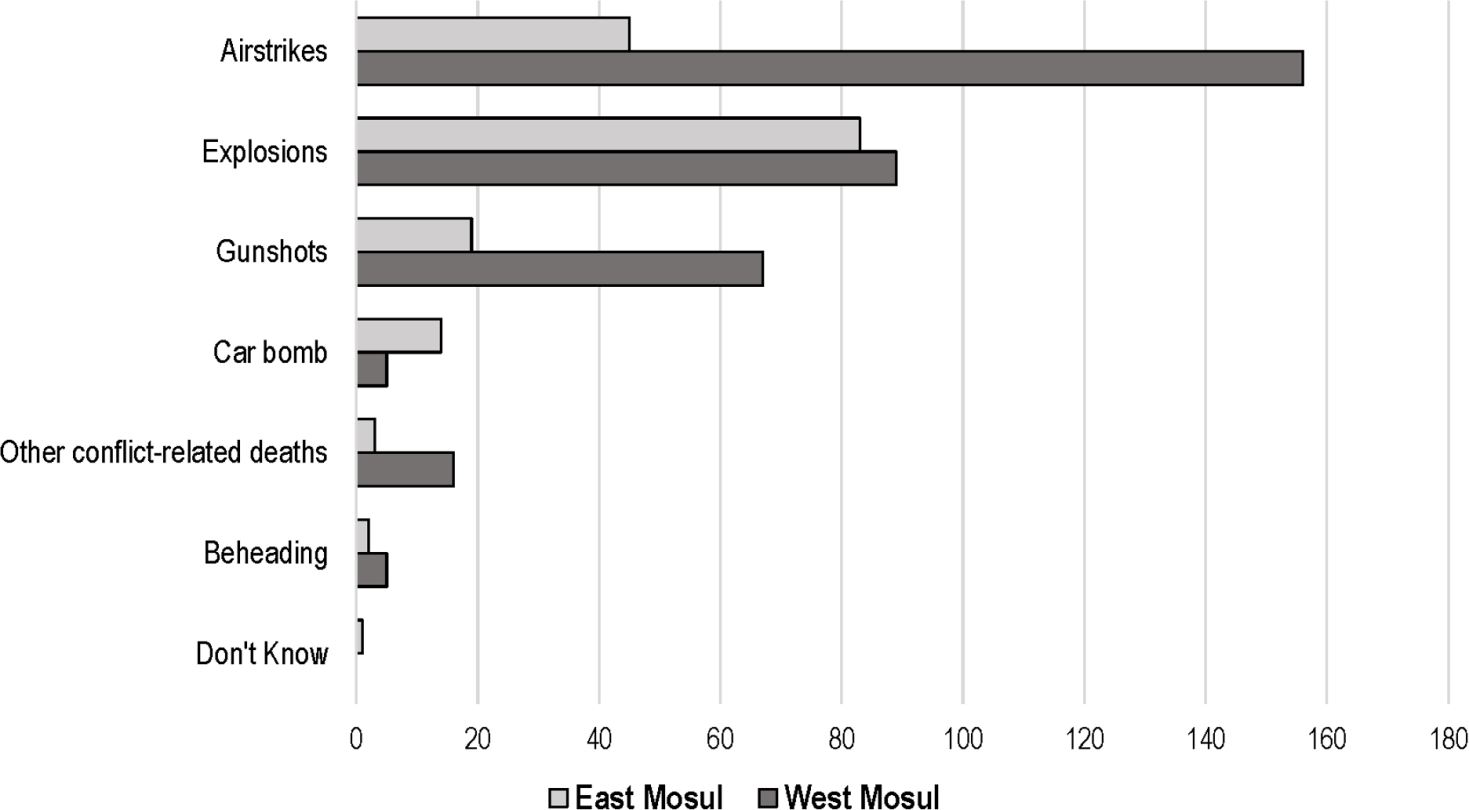
Reported causes of death for east and west Mosul.

The reported causes of death from both nonviolent causes and from intentional violence are shown in Table 4. The most common medical cause of death was cardiovascular disease followed by renal conditions and injuries not related to conflict. Intentional violence was responsible for 338 (84.9%) reported deaths in west Mosul and 167 (74.9%) deaths in east Mosul. In west Mosul survey households, airstrikes were the single most common reported cause of death, accounting for 167 (72.6%) deaths due to intentional violence. In the east Mosul households, airstrikes were responsible for 45 (26.9%) deaths, with explosions the largest single group, responsible for 83 (49.7%) deaths (Fig 5).

**Table 4:** Causes of death and injury.

	East (left) Mosul						West (right) Mosul						Overall					
	Male		Female		Combined		Male		Female		Combined		Male		Female		Combined	
	(*N* = 2,437)		(*N* = 2,430)		(*N* = 4,867)		(*N* = 1,341)		(*N* = 1,351)		(*N* = 2,692)		(*N* = 3,778)		(*N* = 3,781)		(*N* = 7,559)	
	*N*	(%)	*N*	(%)	*N*	(%)	*N*	(%)	*N*	(%)	*N*	(%)	*N*	(%)	*N*	(%)	*N*	(%)
**Deaths**	** **	** **	** **	** **	** **	** **	** **	** **	** **	** **	** **	** **	** **	** **	** **	** **	** **	** **
**Reported nonintentional-violence deaths**					** **	** **	** **	** **	** **	** **	** **	** **	** **	** **	** **	** **	** **	** **
% of all deaths that were not from intentional violence	33	(23.7%)	30	(33.0%)	63	(27.4%)	37	(15.2%)	23	(14.8%)	60	(15.1%)	70	(18.3%)	53	(21.5%)	123	(19.6%)
Cardiovascular	6	(18.2%)	13	(43.3%)	19	(30.2%)	18	(48.6%)	6	(26.1%)	24	(40.0%)	24	(34.3%)	19	(35.8%)	43	(35.0%)
Kidney condition	2	(6.1%)	3	(10.0%)	5	(7.9%)	9	(24.3%)	3	(13.0%)	12	(20.0%)	11	(15.7%)	6	(11.3%)	17	(13.8%)
Cancer or tumor	4	(12.1%)	1	(3.3%)	5	(7.9%)	2	(5.4%)	4	(17.4%)	6	(10.0%)	6	(8.6%)	5	(9.4%)	11	(8.9%)
Injury (not conflict related)	9	(27.3%)	1	(3.3%)	10	(15.9%)	1	(2.7%)	0	(0.0%)	1	(1.7%)	10	(14.3%)	1	(1.9%)	11	(8.9%)
Lung disease	2	(6.1%)	2	(6.7%)	4	(6.3%)	1	(2.7%)	1	(4.3%)	2	(3.3%)	3	(4.3%)	3	(5.7%)	6	(4.9%)
Respiratory	3	(9.1%)	1	(3.3%)	4	(6.3%)	1	(2.7%)	0	(0.0%)	1	(1.7%)	4	(5.7%)	1	(1.9%)	5	(4.1%)
Neonatal causes	2	(6.1%)	1	(3.3%)	3	(4.8%)	0	(0.0%)	2	(8.7%)	2	(3.3%)	2	(2.9%)	3	(5.7%)	5	(4.1%)
Liver disease	2	(6.1%)	1	(3.3%)	3	(4.8%)	1	(2.7%)	0	(0.0%)	1	(1.7%)	3	(4.3%)	1	(1.9%)	4	(3.3%)
Maternal causes	0	(0.0%)	2	(6.7%)	2	(3.2%)	0	(0.0%)	1	(4.3%)	1	(1.7%)	0	(0.0%)	3	(5.7%)	3	(2.4%)
Diarrhea	0	(0.0%)	0	(0.0%)	0	(0.0%)	0	(0.0%)	2	(8.7%)	2	(3.3%)	0	(0.0%)	2	(3.8%)	2	(1.6%)
Preterm births	2	(6.1%)	0	(0.0%)	2	(3.2%)	0	(0.0%)	0	(0.0%)	0	(0.0%)	2	(2.9%)	0	(0.0%)	2	(1.6%)
Other	0	(0.0%)	2	(6.7%)	2	(3.2%)	1	(2.7%)	0	(0.0%)	1	(1.7%)	1	(1.4%)	2	(3.8%)	3	(2.4%)
Don’t know	1	(3.0%)	3	(10.0%)	4	(6.3%)	3	(8.1%)	4	(17.4%)	7	(11.7%)	4	(5.7%)	7	(13.2%)	11	(8.9%)
**Totals**	**33**	** **	**30**	** **	**63**	** **	**37**	** **	**23**	** **	**60**	** **	**70**	** **	**53**	** **	**123**	** **
**Reported intentional-violence deaths**			** **	** **	** **	** **	** **	** **	** **	** **	** **	** **	** **	** **	** **	** **	** **	** **
Airstrike	25	(23.6%)	20	(32.8%)	45	(26.9%)	81	(39.3%)	75	(56.8%)	156	(46.2%)	106	(34.0%)	95	(49.2%)	201	(39.8%)
Explosions	58	(54.7%)	25	(41.0%)	83	(49.7%)	68	(33.0%)	21	(15.9%)	89	(26.3%)	126	(40.4%)	46	(23.8%)	172	(34.1%)
Gunshot	11	(10.4%)	8	(13.1%)	19	(11.4%)	42	(20.4%)	25	(18.9%)	67	(19.8%)	53	(17.0%)	33	(17.1%)	86	(17.0%)
Car bomb	7	(6.6%)	7	(11.5%)	14	(8.4%)	4	(1.9%)	1	(0.8%)	5	(1.5%)	11	(3.5%)	8	(4.1%)	19	(3.8%)
Beheading	2	(1.9%)	0	(0.0%)	2	(1.2%)	5	(2.4%)	0	(0.0%)	5	(1.5%)	7	(2.2%)	0	(0.0%)	7	(1.4%)
Other conflict-related deaths	3	(2.8%)	0	(0.0%)	3	(1.8%)	6	(2.9%)	10	(7.6%)	16	(4.7%)	9	(2.9%)	10	(5.2%)	19	(3.8%)
Don’t know	0	(0.0%)	1	(1.6%)	1	(0.6%)	0	(0.0%)	0	(0.0%)	0	(0.0%)	0	(0.0%)	1	(0.5%)	1	(0.2%)
**Totals**	**106**	** **	**61**	** **	**167**	** **	**206**	** **	**132**	** **	**338**	** **	**312**	** **	**193**	** **	**505**	** **
**Reported causes of injuries from intentional violence**									** **	** **	** **	** **	** **	** **	** **	** **	** **	** **
Shell injuries/fragments	21	47.7%	12	37.5%	33	43.4%	44	57.9%	24	35.3%	68	47.2%	65	54.2%	36	36.0%	101	45.9%
Blast/explosive injury	16	36.4%	17	53.1%	33	43.4%	27	35.5%	36	52.9%	63	43.8%	43	35.8%	53	53.0%	96	43.6%
Gunshot	3	6.8%	2	6.2%	5	6.6%	4	5.3%	6	8.8%	10	6.9%	7	5.8%	8	8.0%	15	6.8%
Burns	0	0.0%	1	3.1%	1	1.3%	1	1.3%	1	1.5%	2	1.4%	1	0.8%	2	2.0%	3	1.4%
Torture (prisoners)	3	6.8%	0	0.0%	3	3.9%	0	0.0%	0	0.0%	0	0.0%	3	2.5%	0	0.0%	3	1.4%
Other	1	2.3%	0	0.0%	1	1.3%	0	0.0%	1	1.5%	1	0.7%	1	0.8%	1	1.0%	2	0.9%
***Totals***	**44**	** **	**32**	** **	**76**	** **	**76**	** **	**68**	** **	**144**	** **	**120**	** **	**100**	** **	**220**	** **

The all-cause mortality rates during the 29 months of exclusive ISIS control and the nine months of the military offensive are shown in person-months in Table 5. The same data are shown in person-years in S2 Table. During ISIS control, mortality rates among males were 0.71 deaths per 1,000 person-months (8.47 per 1,000 person-years) and for females 0.50 deaths per 1,000 person-months (5.99 per 1,000 person-years). During the military offensive, the rates jumped to 13.36 for males (160.4 per 1,000 person-years) and to 8.33 for females (99.92 per 1,000 person-years). The increase in death rates was particularly dramatic in west Mosul, where it increased from a rate of 0.64 to 15.54 deaths per 1,000 person-months (7.71 per 1,000 person-years to 186.5) with the military offensive.

**Table 5:** Death rates by time period (ISIS control and liberation).*

	East Mosul			West Mosul			Overall		
	Total person-months exposed	Total deaths	Rate	Total person-months exposed	Total deaths	Rate	Total person-months exposed	Total deaths	Rate
**ISIS control (July 2014–September 2016)**									
Male	63,821	38	0.60	33,960	31	0.91	97,781	69	0.71
Female	63,710	36	0.57	34,487	13	0.38	98,197	49	0.50
** Combined**	**127,531**	**74**	**0.58**	**68,447**	**44**	**0.64**	**195,978**	**118**	**0.60**
**Liberation (October 2016–March/July 2017)**									
Male	11,694	94	8.04	10,980	209	19.03	22,674	303	13.36
Female	11,823	54	4.57	11,475	140	12.20	23,298	194	8.33
** Combined**	**23,517**	**148**	**6.29**	**22,455**	**349**	**15.54**	**45,972**	**497**	**10.81**

*Deaths rates in persons-years are found in S2 Table.

Abbreviation: ISIS, Islamic State of Iraq and Syria.

### Kidnapping

Households reported that 35 members had been kidnapped during the time of ISIS control, 34 of whom were males. Twenty of these had been the head of household and 15 had been a son. Of the 35 reportedly kidnapped, 7 were still missing, 20 had been released, and 8 were dead.

### Injuries

Households were asked about injuries that occurred to household members during the years of ISIS control and the liberation. The 223 injuries reported in detail were due to intentional violence in all but 3 instances (Table 3). The reported causes of the injuries from intentional violence are shown in Table 4, the majority being shell (45.9%) or blast (43.6%) injuries. Explosions and shrapnel were the main causes of the injuries noted in Table 4. The treatment and status of persons injured from all causes are noted in S3 Table. This lists the treatment received at the time of the injury and status of the injured at the time of the survey. Among the injured, 30 (13.5%) were alive and functioning normally at the time of interview. There were 102 (45.7%) who were alive but with limited function, 7 (3.1%) who had died, and 84 (37.7%) who were convalescing and still receiving treatment. At the time of injury, 153 (68.7%) had received inpatient hospital care. No formal care was reported for the day of injury for 46, or 20.8%, of those injured.

### Physical location of injuries

**Fig 6:**
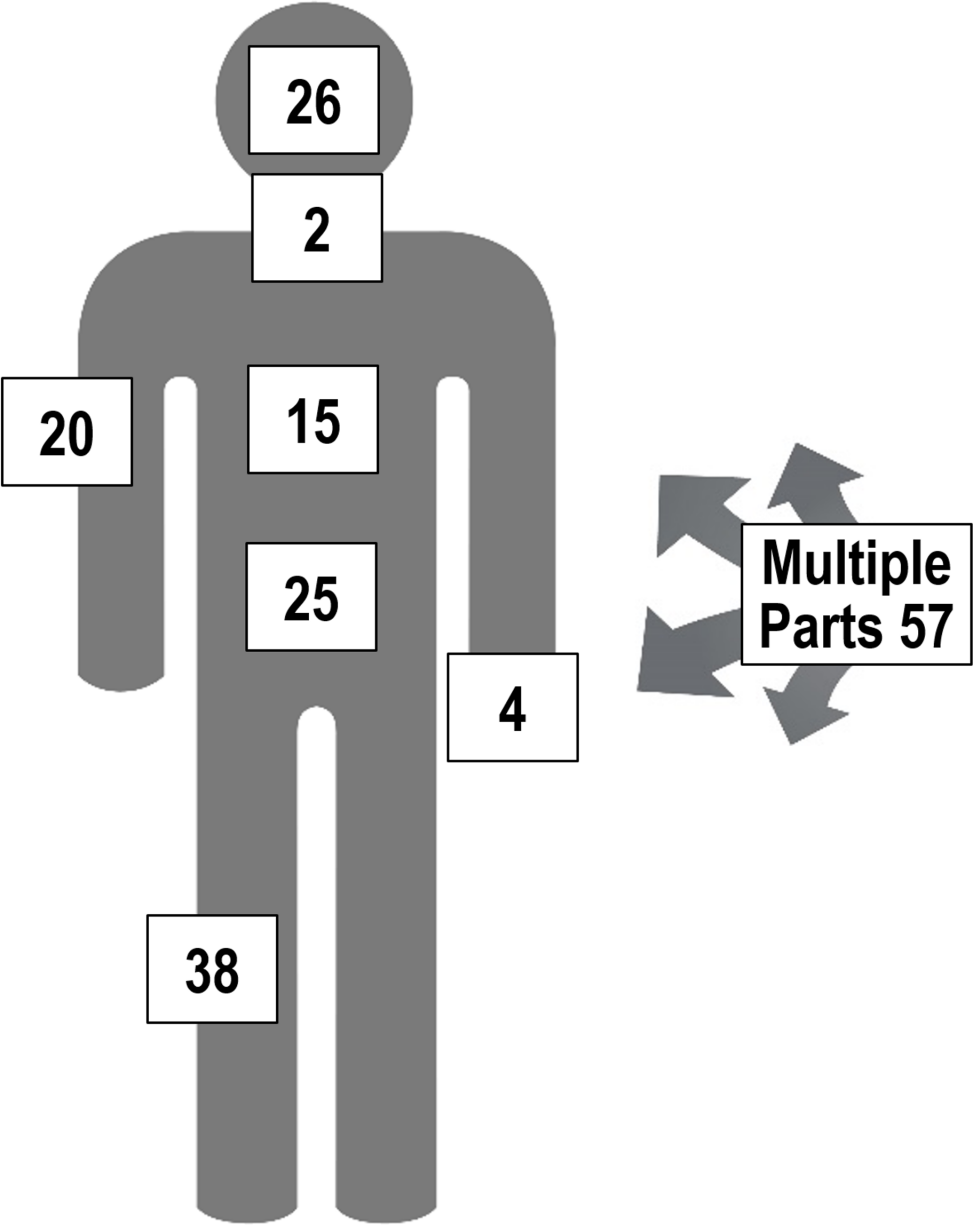
Location of wounds from intentional injuries.

The location of the injuries sustained by persons both in east and west Mosul are shown in Fig 6. Of the 220 injuries noted, the specific location was not recorded for 23 injuries.

## Discussion

This survey from the households of Mosul presents a picture of death and injury, most pronounced during the nine-month military campaign to drive ISIS fighters from the city. The mortality rate per 1,000 person-months jumped from a rate of 0.58 deaths per 1,000 person-months during the 29 months of exclusive ISIS control to a rate of 6.29 in east Mosul during the nine months of liberation. In west Mosul, the numbers increased from an ISIS rate of 0.64 to a rate of 15.54 during the nine months of the military liberation. However, the true numbers of deaths and injuries that occurred in Mosul during this time cannot be known. In some west Mosul clusters, the survey team found nearly as many empty or destroyed dwellings as occupied ones, indicating a large survivor bias potential. These clusters were likely to have sustained a much larger loss of life than surviving households reported. The actual population of Mosul in the time of ISIS and during the military offensive is not known, making it not possible to estimate the numbers of dead and injured for the city as a whole. The study was conducted immediately after ISIS had been driven from east Mosul and then again in west Mosul as soon as it had been secured. The households included had been present in Mosul during the entire 29 months of ISIS control and during the nine months of Iraqi military action. The population characteristics themselves create a remarkable pyramid. Teenage and younger women were underrepresented, most likely related to an increased rate of early marriages driven by the fear that daughters would be forced to marry ISIS fighters, a common situation in conflicts [13]. It is likely that after marriage they left Mosul, either escaping entirely or moving to one of the small towns or villages surrounding Mosul. Persons aged 50 and above constituted 19.3% of the sample, compared with an estimated 10.7% for the Iraqi population as a whole [14]. The percentage of older persons was higher in west Mosul (22.0%) compared with east Mosul (17.8%). Although west Mosul was the older and poorer part of the city, the population pattern suggests that younger persons there may have been better able to flee the battlefront as it moved from east to west. Although some may have managed to escape the city, others fled across the Tigris River into east Mosul as the nature of the final struggle with ISIS became evident.

Until about age 20, death rates from intentional violence for males and females were similar. After age 20, the mortality numbers and rates rose among males but stayed flat among females. At older ages, this difference between males and females narrowed somewhat. This is likely related to increased time outside the household by younger and early middle-aged males, increasing exposure to violent events. These findings demonstrate a pattern similar to other Iraq mortality surveys [15]. Mortality rates from intentional violence were higher in west Mosul (*P* < 0.001) and higher at all ages.

Interviewers recorded the nature of the intentional violence–causing deaths reported by households. In some cases, it may have been difficult for households to differentiate among artillery, mortars, mines, and improvised rocket-assisted munitions (IRAMs), so these were all classified as explosions. Explosions caused 172 deaths, which were in approximately equal numbers in east and west Mosul. The difficulty in precise targeting with these weapons, including heavy artillery fired from 10 to 20 km away, may have contributed to the number of civilian deaths. Airstrikes caused the largest number of household deaths (201), and these were predominantly in west Mosul. Bombs of 500 pounds or greater have a blast radius possibly well beyond the specific target. Even smaller munitions with a narrower blast radius could cause civilian loss of life where ISIS was using civilians as human shields [16]. The Airwars monitoring organization has mapped 1,933 coalition airstrikes in Mosul and in excess of 12,000 airstrikes in Iraq as a whole since the start of 2015 [17]. When targets are examined on the ground, it appears that at times, bombing may have been indiscriminate. Satellite maps show extensive areas of extreme devastation in west Mosul [17]. As the Iraqi forces moved westward across the Tigris, the spike in deaths from the preconflict period followed the movement of the battlefront. The intensity of the conflict in west Mosul caused more household deaths compared with east Mosul, even though the population of the households sampled was smaller. Although military planners claimed that these airstrikes are “one of the most precise air campaigns in military history,” the intensity of the urban conflict is probably unmatched since the Second World War [18]. While some civilian deaths occurred in proximity to legitimate targets—accepted as “necessity” under international humanitarian law (IHL)—many civilians were killed when targets of dubious military importance were targeted, perhaps through faulty or outdated intelligence [19]. The civilian deaths from poor intelligence may have been compounded through a breach of the IHL principle of proportionality, particularly with the use of large ordnance [20]. Moving from the mortality rates found from these 1,202 households to overall estimated for Mosul would be difficult. The events during the time of ISIS control might be assumed to be fairly uniform across the city, so application of rates to an estimated Mosul population could be appropriate. During the eight months of military operations, however, the use of high-powered ordnance was concentrated in certain areas, particularly in certain sectors of west Mosul, while strikes in east Mosul were relatively light. This would make wider population estimates of mortality for Mosul more uncertain.

Of the 220 injuries reported, most were conflict related. The number of nonconflict injuries seems rather small considering that nonconflict injuries were the preponderant injuries reported in our Baghdad injury study [21]. Mosul violent injuries were more likely to involve the abdomen and lower leg than those found in the 2003 to 2014 Baghdad injury study. However, with the high-intensity conflict in Mosul, it is likely that minor nonconflict injuries might be easily forgotten. The time trends associated with injuries for east and west Mosul followed the same pattern as for deaths. It is likely that the causes were the same. The fact that most injuries were treated as hospital inpatients suggests that these were generally of a serious nature. For the 20.8% of those injured who did not receive treatment on the day of injury, this may reflect the lack of treatment facilities more than the severity of the injuries, and it is likely that some died being unable to receive care. At the time of the interview, only 13% of injured persons reported normal function, with over one-third still receiving some form of treatment for their injuries. While recall bias does complicate injury surveys, it is unlikely that serious injuries, especially those resulting in disabilities, are easily forgotten.

Kidnapping was common, though not as extensive as—and occurring for different reasons than—among the Yazidis of Mt. Sinjar or in Baghdad, where kidnapping was politically or criminally motivated [3]. Almost all persons reportedly kidnapped in this survey were males. The sole woman kidnapped was operating a beauty salon for women in her home. Kidnappings occurred before liberation and were related to a person’s previous activities, such as having been a parliamentary candidate or associated with the Iraqi army in some way. Other reasons for kidnapping were the trading of cigarettes or possibly using a mobile phone to contact Iraq forces. No kidnapped person escaped; all were either released or were killed. At the time of the survey, there were some who were still missing.

The distribution of causes of death not attributed to intentional violence is what might be expected of an Iraqi population. However, health workers felt that lack of access to specialist diagnosis and treatment, shortage of medicines, and limited access to food may have contributed to a higher mortality in some conditions.

These data illustrate what can be learned about illness and death in conflict situations as well as the limitations of surveys. They also illustrate the additional measures required as well as the personal risks in these types of surveys, which are absent in stable situations. Our findings should be viewed as providing an important, though necessarily an incomplete, picture of events. The final assessment of the state of the population during the 29 months of ISIS control and the nine months of the liberation conflict will come from the triangulation of the findings presented here with other data, both qualitative and quantitative.

### Limitations

There are many limitations to this study, which was designed to assess population trends and patterns from seizure by ISIS to the liberation by Iraqi forces, some 37 months later. The true population of Mosul during the time of ISIS and during the liberation is unknown. Although we used pre-ISIS population distribution data for Mosul, there is no assurance that this distribution remained accurate at the time of survey. Particularly in west Mosul, the population numbers and distribution were difficult to estimate because much of the housing was destroyed by airstrikes and explosives. We tried to compensate for this by dropping neighborhoods that were extensively damaged or otherwise depopulated; however, this may not have been sufficient. Survivor bias—that is, the inability to collect information from households destroyed—undoubtedly was an important limitation. Some households were able to flee Mosul and thus were not counted. Other households were entombed by artillery airstrikes and from the actions of ISIS, which has been verified during excavations in damaged parts of Mosul [22]. This survey represented the survivors, so it could not be an entirely cross-sectional sample of the population who lived in Mosul during ISIS control. The large number of empty or destroyed houses in west Mosul clusters most certainly indicates a substantial underestimation of mortality and injury. Household members may have had difficulty differentiating artillery explosions from airstrikes. Because neighborhoods are often family neighborhoods, survivors from destroyed households could join with relatives from other households, and thus the deaths and injuries reported might be incorrectly attributed to the household being interviewed, though in these cases separate kitchens were usually maintained. Divided households might have specific events double counted. Although we tried to conservatively estimate the person-months of exposure, it is possible that there was a flux of household residents who were not accurately counted.

### Conclusion

Despite many limitations, these data depict a city heavily damaged. Although the media accounts report extensive brutality and wanton killing by ISIS, we found that the military ground and airstrikes resulted in a death toll exceeding that reported during the ISIS occupation. The description of airstrikes that were stated to be “one of the most precise air campaigns in military history” seems not to be supported by our data, which found large numbers of civilian deaths attributed to airstrikes [19]. The military offensive brought an increase in injury and death that affected the young, the robust, and the elderly of Mosul to varying degrees. Some persons were able to escape the city or escape the destruction around them, but many were not, and their number we cannot completely measure.

## Supporting information

S1 STROBE Checklist STROBE statement. (DOC)

S1 Text Mosul household questionnaire (injury and mortality sections). (PDF)

S1 Table Death and injury rates for west and east Mosul with IRRs, 20,091 person-years exposure. IRR, incidence rate ratio.
(DOC)

S2 Table Death rates in person-years by time period (ISIS control and liberation). ISIS, Islamic State of Iraq and Syria.
(DOC)

S3 Table Treatment and status of persons injured (all causes). (DOCX)

## Abbreviations

IDP: internally displaced person
IHL: international humanitarian law
IRAM: improvised rocket-assisted munition
IRR: incidence rate ratio
ISF: Iraqi security forces
ISIS: Islamic State of Iraq and Syria
PMU: Popular Militia Unit
STROBE: Strengthening the Reporting of Observational Studies in Epidemiology
