# Supplementary material for: Injury and death during the ISIS occupation of Mosul and its liberation: Results from a 40-cluster household survey
Source: PLoS Med. 2018 May 15;15(5):e1002567. doi: 10.1371/journal.pmed.1002567 (PMC5953440; doi:10.1371/journal.pmed.1002567)
Supplement: S1 STROBE Checklist — (DOC) [file pmed.1002567.s001.doc]

***S1. STROBE Statement****—Checklist for Injury and death during the ISIS occupation of Mosul and its liberation: Results from a 40-cluster household survey*

|  | Item No | Recommendation |
| --- | --- | --- |
| **Title and abstract** | 1 | (*a*) Indicate the study’s design with a commonly used term in the title or the abstract  *Manuscript title* |
|  |  | (*b*) Provide in the abstract an informative and balanced summary of what was done and what was found  *Abstract seeks to provide a balanced picture of methods, results and conclusion* |
| Introduction | | |
| Background/rationale | 2 | Explain the scientific background and rationale for the investigation being reported  *Measurement of mortality in conflict situation presents many challenges, yet in situations such as ISIS control of Mosul and military actions, population based sampling represent and established approach to household measurements.* |
| Objectives | 3 | State specific objectives, including any prespecified hypotheses  *The survey objectives were to measure deaths, injuries and kidnapping during the 29 months of exclusive control by Islamic State of Iraq and Syria (ISIS), and during the eight months of Iraqi military action (known as the liberation).*PARA 5 |
| Methods | | |
| Study design | 4 | Present key elements of study design early in the paper.  *Forty neighborhoods or administrative units were randomly selected from Mosul’s established residential administrative units, 25 to the east of the Tigris river and 15 to the west. Thirty households were sampled in each neighbourhood. PARA 1* |
| Setting | 5 | Describe the setting, locations, and relevant dates, including periods of recruitment, exposure, follow-up, and data collection  *East (March 2017) and west Mosul (July 2017). Data were collected from the selected neighbourhoods by four data collectors during approximately 4 weeks PARA 5* |
| Participants | 6 | (*a*) Give the eligibility criteria, and the sources and methods of selection of participants  *An inclusion requirement was that a household had been present during the entire period from June 2014. PARA 3, 4* |
| Variables | 7 | Clearly define all outcomes, exposures, predictors, potential confounders, and effect modifiers. Give diagnostic criteria, if applicable  *Deaths, injuries and kidnapping during the 29 months of exclusive control by Islamic State of Iraq and Syria (ISIS), and during the eight months of Iraqi military action. PARA 2* |
| Data sources/ measurement | 8 | For each variable of interest, give sources of data and details of methods of assessment (measurement). Describe comparability of assessment methods if there is more than one group*. Source of data were 1202 households who had been living in east or west Mosul during the period of ISIS control and during the military liberation. PARA 1* |
| Bias | 9 | Describe any efforts to address potential sources of bias.  *We acknowledge the many potential sources of bias. Survivor bias is one of the greatest, we replaced two neighborhoods where there was insecurity or a high number of unoccupied or destroyed dwellings. PARA 6* |
| Study size | 10 | Explain how the study size was arrived at  *Based on previous Iraqi studies, 1200 households would give both good demographic data and be representative of a large urban area. Yet risks to interviewers for this sample size were felt acceptable to the interviewers. PARA 2* |
| Quantitative variables | 11 | Explain how quantitative variables were handled in the analyses. If applicable, describe which groupings were chosen and why  *exposure times reflect the month of entry or exit from the household, notably in the case of death, kidnapping, or birth. Incidence rates for death and injury were calculated from the total number of deaths/injuries divided by the total number of person-months. Stratified incidence rates were calculated in the same manner individually by age group, geographic area (west/east Mosul), sex, and time period (ISIS occupation vs. liberation).PARA 7* |
| Statistical methods | 12 | (*a*) Describe all statistical methods, including those used to control for confounding  (*b*) Describe any methods used to examine subgroups and interactions  (*c*) Explain how missing data were addressed  (*d*) If applicable, describe analytical methods taking account of sampling strategy  (*e*) Describe any sensitivity analyses  *Data were analysed using Stata,. Incidence rates for death and injury were calculated from the total number of deaths/injuries divided by the total number of person-months contributed using the ‘stptime’ command in Stata, which allowed for variable follow-up time for each individual by dividing the number of failures.* *Stratified incidence rates were calculated in the same manner individually by age group, geographic area (west/east Mosul), sex, and time period (ISIS occupation vs. liberation). For each of these analyses, the number of deaths or injuries in each respective age, sex, area group, or time period was divided by the total number of person-time contributed, then multiplied to estimate rates per 1,000 person-months. To calculate incidence rate ratios comparing death rates between geographic areas (in west versus /east Mosul) within sex and age groups, a Poisson model was fit allowing person-specific time contributed (in person-months). Bootstrapping was used to account for clustering that occurred at the sampling level in calculating confidence intervals. PARA 7* |
| Results | | |
| Participants | 13 | (a) Report numbers of individuals at each stage of study—eg numbers potentially eligible, examined for eligibility, confirmed eligible, included in the study, completing follow-up, and analysed  *There were a total of 7559 persons from the 1202 households in the study, 4867 from east Mosul and 2692 from west Mosul. PARA 1* |
|  |  | (b) Give reasons for non-participation at each stage  *There were no refusals to participate, a common finding in conflict-area surveys* |
| Descriptive data | 14 | (a) Give characteristics of study participants (eg demographic, clinical, social) and information on exposures and potential confounders.  *These were 7559 persons living in 1202 households in Mosul. Household size varied form 6.0-6.5 persons. All households included had been present in Mosul during the entire period of control by ISIS and during the military campaign to free Mosul. PARA 2 Table 2* |
|  |  | (b) Indicate number of participants with missing data for each variable of interest  *Missing data were not a problem in this study. However households many have chosen not to report various events. Survivor bias is acknowledged to be a major problem. PARA 1* |
| Outcome data | 15 | Report numbers of outcome events or summary measures  *Deaths and injuries by age and sex as well as causes of death are reported in Tables 3-5* |
| Main results | 16 | (*a*) Give unadjusted estimates and, if applicable, confounder-adjusted estimates and their precision (eg, 95% confidence interval). Make clear which confounders were adjusted for and why they were included  *Estimates are not included in this paper* |
|  |  | (*b*) Report category boundaries when continuous variables were categorized  *In Tables 3-5 ages are included as well as sex and location* |
| Other analyses | 17 | Report other analyses done—eg analyses of subgroups and interactions, and sensitivity analyses  *The types of injuries are reported in results and the medical management of the injuries are noted in the supporting information* |
| Discussion | | |
| Key results | 18 | Summarise key results with reference to study objective  *Information relating to events under ISIS, and during the liberation are summarized by age and sex as well as location and type of event. PARA 1* |
| Limitations | 19 | Discuss limitations of the study, taking into account sources of potential bias or imprecision. Discuss both direction and magnitude of any potential bias  *There are many potential limitations to this study in a population traumatized by conflict and these are noted in the discussion. PARA 8* |
| Interpretation | 20 | Give a cautious overall interpretation of results considering objectives, limitations, multiplicity of analyses, results from similar studies, and other relevant evidence  *The impact of life during ISIS control and during the military liberation are discussed. PARA 9* |
| Generalisability | 21 | Discuss the generalisability (external validity) of the study results  *The implication of warfare with high intensity ordnance in densely population urban areas are discussed. PARA 4* |
| Other information | | |
| Funding | 22 | Give the source of funding and the role of the funders for the present study and, if applicable, for the original study on which the present article is based.  *The use of only internal funding is stated.* |
