# Supplementary material for: Injury and death during the ISIS occupation of Mosul and its liberation: Results from a 40-cluster household survey
Source: PLoS Med. 2018 May 15;15(5):e1002567. doi: 10.1371/journal.pmed.1002567 (PMC5953440; doi:10.1371/journal.pmed.1002567)
Supplement: S1 Text — (PDF) [file pmed.1002567.s002.pdf]

S2. Mosul household questionnaire (Injury and mortality section)

Date_________ Interviewer code______

Cluster No_____ Family No. _____ Is this a replacement Household? ☐ YES ☐ NO

Has household consented? ☐ YES ☐ NO *if no please stop unless consented*

1. Has this household been continuously present in Mosul since June 2014 ☐ YES ☐ NO *if no please stop*

2. Present in the household from June 2014

Given name	age	sex	Education code	Employment code	Injury since June 2014 Y/N	Missing since June 2014 Y/N	Death since June 2014 Y/N
1.House head							
2.							
3.							
4.							
5.							
6							
7.							
8							
9							
10.							
11.							
12							
			1=none<br>2=in school<br>3=primary school<br>4=secondary school<br>5=post secondary	1=works full time<br>2=works part time<br>3=unemployed<br>4=student<br>5=housewife<br>6=retired		1=Kidnapped<br>2=presumed to be alive in Mosul<br>3=presumed to be alive outside Mosul<br>4=probably dead<br>5=unknown	

3. Since JUNE 2014, have there been any DEATHS among adults or children who were living in this household?

For questions (f) and (h) select the choice that most closely matches the description provided by the person—Do not read out the options.

Death no. (a)	Sex M/F (b)	Date of death? month & year (c)	Age at death? (d)	Relation to key informant (e)	What was the cause of death? (f)	Was death conflict related? Y/N/DK (g)	*If conflict-related,* What was the cause? (h)

(e): 1=wife, 2=husband, 3=son, 4=daughter, 5=mother, 6=father, 7=sister, 8=brother, 9=mother-in-law, 10=father-in-law, 11=sister-in-law, 12=brother-in-law, 13=nephew, 14=niece, 15=grandson, 16=granddaughter, 17=other

(f): 1=diarrhea, 2=respiratory, 3=pre-term, 4=neonatal causes, 5=cancer/tumor, 6=cardiovascular disease, 7=lung disease, 8=liver disease, 9=kidney condition, 10=maternal causes, 11=self-inflected injury, 12=injury (not conflict-related), 13=injury (conflict-related), 14=other, 15=don't know

(h): 1=gunshot, 2=stabbing, 3=beheading, 4=car bomb, 5=road accident that was conflict-related, 6=airstrike, 7=other explosion, 8=other conflict-related injury, 9=don't know

4. Since JUNE 2014, have there been any INJURIES among persons who were living in this household?

Injury Definition: An intentional or unintentional physical event that requires medical care and/or intervention and results in loss or reduction in normal activities for a while For questions (f), (g), (i), and (k) select the choice that most closely matches the description provided by the person—Do not read out the options.

House hold list no. (a)	Sex M/F (b)	Date of injury? month & year (c)	Age at injury ? (d)	Relation to key informant (e)	Parts of the body injured? (more than one possible) (f)	The cause of injury? (g)	Was injury conflict related? Y/N/DK (h)	*If yes,* What was the cause? (i)	*Current status of injured* (j)	*Treatment Received on day of injury (k)*

(e): 1=wife, 2=husband, 3=son, 4=daughter, 5=mother, 6=father, 7=sister, 8=brother, 9=mother-in-law, 10=father-in-law, 11=sister-in-law, 12=brother-in-law, 13=nephew, 14=niece, 15=grandson, 16=granddaughter, 17=other 18=daughter-in-law

(f):1=head, 2=face, 3=neck, 4=chest, 5=back or spine, 6=abdomen, 7=upper extremity (except hand), 8=hand, 9=lower extremity

(g): 1=burn, 2=electrical, 3=fall, 4=poisoning, 5=mechanical (such as industrial trauma), 6=explosion (unintended violence), 7=suicidal intent, 8=transportation related, 9=intentional violence; 10=other, 11=don't know

(i): 1=gunshot, 2=penetrating wounds (including stab wounds), 3=shell injuries/fragment, 4=blast/explosive injury, 7=burns, 8=torture (prisoners), 9=other, 10=don't know

(j): 1=alive and functioning normally, 2=alive with reduced function, 3=dead 4= still under treatment

(k): 1=home treatment, 2=treated in surgery or clinic (outpatient), 3=Admitted to hospital (inpatient), 4=no treatment received

5. Was any member of this household kidnapped during or after June 2014?

Kidnapped no. (a)	Sex M/F (b)	Date of kidnapping? month & year (c)	Age at kidnapping? (d)	Relation to key informant (e)	Current status? (f)

(e): 1=wife, 2=husband, 3=son, 4=daughter, 5=mother, 6=father, 7=sister, 8=brother, 9=mother-in-law, 10=father-in-law, 11=sister-in-law, 12=brother-in-law, 13=nephew, 14=niece, 15=grandson, 16=granddaughter, 17=other 18=Daughter-in-law

(f): 1=still missing, 2=released/escaped, 3=dead
