# Supplementary material for: Injury and death during the ISIS occupation of Mosul and its liberation: Results from a 40-cluster household survey
Source: PLoS Med. 2018 May 15;15(5):e1002567. doi: 10.1371/journal.pmed.1002567 (PMC5953440; doi:10.1371/journal.pmed.1002567)
Supplement: S1 Table — IRR, incidence rate ratio. (DOC) [file pmed.1002567.s003.doc]

**S1 Table. Death and injury rates for west and east Mosul with IRR, 20,091 person-years exposure.**

| **Death and injury rates for west and east Mosul with incidence rate ratios, 20,091 person-years exposure.** | | | | | | | | | | | | | | | | | | |
| --- | --- | --- | --- | --- | --- | --- | --- | --- | --- | --- | --- | --- | --- | --- | --- | --- | --- | --- |
| **Deaths** |  |  |  |  |  |  |  |  |  |  |  |  |  |  |  |  |  |  |
| **All**   **causes** | |  |  |  |  |  |  |  |  |  |  |  |  |  |  |  |  |  |
|  | **West Mosul** | | | | | | **East Mosul** | | | | | | **Overall** | | | | | |
|  | **Males** | | | **Females** | | | **Males** | | | **Females** | | | **Males** | | | **Females** | | |
| **Age** | **Person years** | **Deaths** | **Rate** | **Person years** | **Deaths** | **Rate** | **Person years** | **Deaths** | **Rate** | **Person years** | **Deaths** | **Rate** | **Person years** | **Deaths** | **Rate** | **Person years** | **Deaths** | **Rate** |
| < 5 | 332 | 15 | 45.24 | 357 | 25 | 70.08 | 690 | 9 | 13.05 | 734 | 9 | 12.26 | 1,021 | 24 | 23.5 | 1,091 | 34 | 31.17 |
| 5 - 9 | 1,072 | 40 | 37.33 | 1,245 | 38 | 30.52 | 2,062 | 33 | 16 | 1,912 | 27 | 14.12 | 3,134 | 73 | 23.3 | 3,157 | 65 | 20.59 |
| 20-39 | 1,200 | 92 | 76.65 | 919 | 37 | 40.28 | 1,922 | 48 | 24.98 | 1,892 | 26 | 13.74 | 3,122 | 140 | 44.84 | 2,811 | 63 | 22.41 |
| 40-59 | 731 | 42 | 57.46 | 921 | 30 | 32.57 | 1,133 | 21 | 18.54 | 1,306 | 7 | 5.36 | 1,864 | 63 | 33.81 | 2,228 | 37 | 16.61 |
| 60+ | 391 | 54 | 138.28 | 351 | 25 | 71.33 | 476.1667 | 28 | 58.8 | 447 | 22 | 49.19 | 867 | 82 | 94.62 | 798 | 47 | 58.92 |
| ***Total*** | 3,725 | 243 | 65.23 | 3,792 | 155 | 40.87 | 6,282 | 139 | 22.13 | 6,292 | 91 | 14.46 | 10,007 | 382 | 38.17 | 10,084 | 246 | 24.4 |
| **Intentional violence** | |  |  |  |  |  |  |  |  |  |  |  |  |  |  |  |  |  |
|  | **West Mosul** | | | | | | **East Mosul** | | | | | | **Overall** | | | | | |
|  | **Males** | | | **Females** | | | **Males** | | | **Females** | | | **Males** | | | **Females** | | |
| **Age** | **Person years** | **Deaths** | **Rate** | **Person years** | **Deaths** | **Rate** | **Person years** | **Deaths** | **Rate** | **Person years** | **Deaths** | **Rate** | **Person years** | **Deaths** | **Rate** | **Person years** | **Deaths** | **Rate** |
| < 5 | 332 | 15 | 45.24 | 357 | 24 | 67.27 | 690 | 6 | 8.7 | 734 | 7 | 9.54 | 1,021 | 21 | 20.56 | 1,091 | 31 | 28.42 |
| 5 - 9 | 1,072 | 40 | 37.33 | 1,245 | 34 | 27.31 | 2,062 | 32 | 15.52 | 1,912 | 25 | 13.08 | 3,134 | 72 | 22.98 | 3,157 | 59 | 18.69 |
| 20-39 | 1,200 | 90 | 74.98 | 919 | 34 | 37.02 | 1,922 | 43 | 22.38 | 1,892 | 23 | 12.15 | 3,122 | 133 | 42.6 | 2,811 | 57 | 20.28 |
| 40-59 | 731 | 40 | 54.72 | 921 | 28 | 30.4 | 1,133 | 14 | 12.36 | 1,306 | 5 | 3.83 | 1,864 | 54 | 28.98 | 2,228 | 33 | 14.81 |
| 60+ | 391 | 21 | 53.78 | 351 | 12 | 34.24 | 476.1667 | 11 | 23.1 | 447.25 | 1 | 2.24 | 867 | 32 | 36.92 | 798 | 13 | 16.3 |
| ***Total*** | 3,725 | 206 | 55.3 | 3,792 | 132 | 34.81 | 6,282 | 106 | 16.87 | 6,292 | 61 | 9.69 | 10,007 | 312 | 31.18 | 10,084 | 193 | 19.14 |
| **Non-intentional violence** | | |  |  |  |  |  |  |  |  |  |  |  |  |  |  |  |  |
|  | **West Mosul** | | | | | | **East Mosul** | | | | | | **Overall** | | | | | |
|  | **Males** | | | **Females** | | | **Males** | | | **Females** | | | **Males** | | | **Females** | | |
| **Age** | **Person years** | **Deaths** | **Rate** | **Person years** | **Deaths** | **Rate** | **Person years** | **Deaths** | **Rate** | **Person years** | **Deaths** | **Rate** | **Person years** | **Deaths** | **Rate** | **Person years** | **Deaths** | **Rate** |
| < 5 | 332 | 0 | 0 | 357 | 1 | 2.8 | 690 | 3 | 4.35 | 734 | 2 | 2.72 | 1,021 | 3 | 2.94 | 1,091 | 3 | 2.75 |
| 5 - 9 | 1,072 | 0 | 0 | 1,245 | 4 | 3.21 | 2,062 | 1 | 0.48 | 1,912 | 2 | 1.05 | 3,134 | 1 | 0.32 | 3,157 | 6 | 1.9 |
| 20-39 | 1,200 | 2 | 1.67 | 919 | 3 | 3.27 | 1,922 | 5 | 2.6 | 1,892 | 3 | 1.59 | 3,122 | 7 | 2.24 | 2,811 | 6 | 2.13 |
| 40-59 | 731 | 2 | 2.74 | 921 | 2 | 2.17 | 1,133 | 7 | 6.18 | 1,306 | 2 | 1.53 | 1,864 | 9 | 4.83 | 2,228 | 4 | 1.8 |
| 60+ | 391 | 33 | 84.51 | 351 | 13 | 37.09 | 476.1667 | 17 | 35.7 | 447.25 | 21 | 46.95 | 867 | 50 | 57.69 | 798 | 34 | 42.62 |
| ***Total*** | 3,725 | 37 | 9.93 | 3,792 | 23 | 6.07 | 6,282 | 33 | 5.25 | 6,292 | 30 | 4.77 | 10,007 | 70 | 7 | 10,084 | 53 | 5.26 |
| **Injuries** |  |  |  |  |  |  |  |  |  |  |  |  |  |  |  |  |  |  |
| **All causes** | |  |  |  |  |  |  |  |  |  |  |  |  |  |  |  |  |  |
|  | **West Mosul** | | | | | | **East Mosul** | | | | | | **Overall** | | | | | |
|  | **Males** | | | **Females** | | | **Males** | | | **Females** | | | **Males** | | | **Females** | | |
| **Age** | **Person years** | **Injuries** | **Rate** | **Person years** | **Injuries** | **Rate** | **Person years** | **Injuries** | **Rate** | **Person years** | **Injuries** | **Rate** | **Person years** | **Injuries** | **Rate** | **Person years** | **Injuries** | **Rate** |
| < 5 | 332 | 2 | 6.03 | 357 | 6 | 16.82 | 690 | 0 | 0 | 734 | 5 | 6.81 | 1,021 | 2 | 1.96 | 1,091 | 11 | 10.08 |
| 5 - 9 | 1,072 | 31 | 28.93 | 1,245 | 23 | 18.47 | 2,062 | 16 | 7.76 | 1,912 | 18 | 9.41 | 3,134 | 47 | 15 | 3,157 | 41 | 12.99 |
| 20-39 | 1,200 | 27 | 22.49 | 919 | 18 | 19.6 | 1,922 | 18 | 9.37 | 1,892 | 6 | 3.17 | 3,122 | 45 | 14.41 | 2,811 | 24 | 8.54 |
| 40-59 | 731 | 13 | 17.78 | 921 | 18 | 19.54 | 1,133 | 9 | 7.95 | 1,306 | 1 | 0.77 | 1,864 | 22 | 11.81 | 2,228 | 19 | 8.53 |
| 60+ | 391 | 4 | 10.24 | 351 | 4 | 11.41 | 476.1667 | 1 | 2.1 | 447.25 | 3 | 6.71 | 867 | 5 | 5.77 | 798 | 7 | 8.77 |
| ***Total*** | 3,725 | 77 | 20.67 | 3,792 | 69 | 18.2 | 6,282 | 44 | 7 | 6,292 | 33 | 5.24 | 10,007 | 121 | 12.09 | 10,084 | 102 | 10.12 |
