# Supplementary material for: Injury and death during the ISIS occupation of Mosul and its liberation: Results from a 40-cluster household survey
Source: PLoS Med. 2018 May 15;15(5):e1002567. doi: 10.1371/journal.pmed.1002567 (PMC5953440; doi:10.1371/journal.pmed.1002567)
Supplement: S2 Table — ISIS, Islamic State of Iraq and Syria. (DOC) [file pmed.1002567.s004.doc]

**S2 Table. Death rates in person years by time period (ISIS control and Liberation).**

|  | **East Mosul** | | | **West Mosul** | | | **Overall** | | |
| --- | --- | --- | --- | --- | --- | --- | --- | --- | --- |
|  | **Total person years exposed** | **Total Deaths** | **Rate** | **Total person years exposed** | **Total Deaths** | **Rate** | **Total person years exposed** | **Total Deaths** | **Rate** |
| **ISIS control (July 2014 - September 2016)** | | | | | | | | | |
| Male | 5,318 | 38 | 7.14 | 2,830 | 31 | 10.95 | 8,148 | 69 | 8.468 |
| Female | 5,309 | 36 | 6.78 | 2,874 | 13 | 4.523 | 8,183 | 49 | 5.988 |
| **Combined** | **10,628** | **74** | **6.96** | **5,704** | **44** | **7.714** | **16,332** | **118** | **7.225** |
| **Liberation (October 2016 - March/July 2017)** | | | | | | | | | |
| Male | 975 | 94 | 96.5 | 915 | 209 | 228.4 | 1,890 | 303 | 160.4 |
| Female | 985 | 54 | 54.8 | 956 | 140 | 146.4 | 1,942 | 194 | 99.92 |
| **Combined** | **1,960** | **148** | **75.5** | **1,871** | **349** | **186.5** | **3,831** | **497** | **129.7** |
