# Supplementary material for: Injury and death during the ISIS occupation of Mosul and its liberation: Results from a 40-cluster household survey
Source: PLoS Med. 2018 May 15;15(5):e1002567. doi: 10.1371/journal.pmed.1002567 (PMC5953440; doi:10.1371/journal.pmed.1002567)
Supplement: S3 Table — (DOCX) [file pmed.1002567.s005.docx]

| **S3 Table. Treatment and status of persons injured (all causes).** | | | | | | | | | | | | |
| --- | --- | --- | --- | --- | --- | --- | --- | --- | --- | --- | --- | --- |
|  | **East Mosul** | | | | **West Mosul** | | | | **Overall** | | | |
|  | **Male** | | **Female** | | **Male** | | **Female** | | **Male** | | **Female** | |
|  | **N** | **(%)** | **N** | **(%)** | **N** | **(%)** | **N** | **(%)** | **N** | **(%)** | **N** | **(%)** |
| **CURRENT STATUS** |  |  |  |  |  |  |  |  |  |  |  |  |
| Alive and functioning normally | 8 | 18.2% | 4 | 12.1% | 8 | 10.4% | 10 | 14.5% | 16 | 13.2% | 14 | 13.7% |
| Alive with reduced function | 24 | 54.5% | 17 | 51.5% | 37 | 48.1% | 24 | 34.8% | 61 | 50.4% | 41 | 40.2% |
| Dead | 0 | (0%) | 0 | (0%) | 4 | 5.2% | 3 | 4.3% | 4 | 3.3% | 3 | 2.9% |
| Still on treatment | 12 | 27.3% | 12 | 36.4% | 28 | 36.4% | 32 | 46.4% | 40 | 33.1% | 44 | 43.1% |
| ***Totals*** | **44** |  | **33** |  | **77** |  | **69** |  | **121** |  | **102** |  |
| **TREATMENT RECEIVED ON THE DAY OF INJURY** |  |  |  |  |  |  |  |  |  |  |  |  |
| Home treatment | 5 | 11.4% | 3 | 9.1% | 0 | 0.0% | 3 | 4.4% | 5 | 4.2% | 6 | 5.9% |
| Treated in surgery or clinic (outpatient) | 8 | 18.2% | 3 | 9.1% | 0 | (0%) | 0 | (0%) | 8 | 6.7% | 3 | 3.0% |
| Admitted to hospital (inpatient) | 25 | 56.8% | 24 | 72.7% | 57 | 75.0% | 47 | 69.1% | 82 | 68.3% | 71 | 70.3% |
| No treatment given | 6 | 13.6% | 3 | 9.1% | 19 | 25.0% | 18 | 26.5% | 25 | 20.8% | 21 | 20.8% |
| ***Totals*** | **44** |  | **33** |  | **76** |  | **68** |  | **120** |  | **101** |  |
